# Antioxidant Defenses in Plants with Attention to *Prunus* and *Citrus* spp.

**DOI:** 10.3390/antiox2040340

**Published:** 2013-11-26

**Authors:** Milvia Luisa Racchi

**Affiliations:** Department of Agri-Food Production and Environmental Sciences, Section of Agricultural Genetics–DISPAA, University of Florence, via Maragliano 77, Firenze 50144, Italy; E-Mail: milvia.racchi@unifi.it; Tel.: +39-055-328-8543; Fax: +39-055-328-8544

**Keywords:** reactive oxygen species, ROS, antioxidant enzymes, antioxidant molecules, *Prunus* spp., *Citrus* spp., ascorbic acid, vitamin C, carotenoids, flavonoids

## Abstract

This short review briefly introduces the formation of reactive oxygen species (ROS) as by-products of oxidation/reduction (redox) reactions, and the ways in which the antioxidant defense machinery is involved directly or indirectly in ROS scavenging. Major antioxidants, both enzymatic and non enzymatic, that protect higher plant cells from oxidative stress damage are described. Biochemical and molecular features of the antioxidant enzymes superoxide dismutase (SOD), catalase (CAT), and ascorbate peroxidase (APX) are discussed because they play crucial roles in scavenging ROS in the different cell compartments and in response to stress conditions. Among the non enzymatic defenses, particular attention is paid to ascorbic acid, glutathione, flavonoids, carotenoids, and tocopherols. The operation of ROS scavenging systems during the seasonal cycle and specific developmental events, such as fruit ripening and senescence, are discussed in relation to the intense ROS formation during these processes that impact fruit quality. Particular attention is paid to *Prunus* and *Citrus* species because of the nutritional and antioxidant properties contained in these commonly consumed fruits.

## 1. Introduction

Antioxidants are compounds produced by aerobic organisms to counteract oxidative stress caused by an imbalance of reactive oxygen species (ROS). ROS include the superoxide anion radicals and the hydroxyl radicals produced as by-products of oxidation/reduction (redox) reactions as a consequence of aerobic metabolism [1].

At the very beginning, the earth’s atmosphere was essentially free of oxygen. Presumably, the most primitive organisms were anaerobic heterotrophs, living protected from solar ionizing radiation damage in the ocean abysses. The earliest, relatively low levels of oxygen probably resulted from photolytic dissociation of water caused by the sun’s ionizing radiation. Later, photosynthetic activities of cyanobacteria and plants steadily boosted oxygen levels, and aerobic metabolism evolved. As a consequence of increasing oxygen levels, all organisms exposed to an aerobic environment are subjected to the toxic effects of ROS.

Molecular oxygen is relatively non-reactive in its normal configuration. During normal metabolic activity and as a consequence of environmental variations, O_2_ can generate reactive excited states such as free radicals and derivatives: the singlet oxygen (^1^O_2_), superoxide radical (O_2_^•−^), hydrogen peroxide (H_2_O_2_), and the hydroxyl radical (OH^•^). The latter is the most potent oxidant known. ROS attack biomolecules causing DNA mutation, protein denaturation and membrane lipid peroxidation; these oxidations disturb normal cellular metabolism and cause molecular damage, and if sufficiently severe can result in cell death.

In plants, the two major sources of ROS are the photosynthetic and respiratory electron transport chains. Chloroplasts and peroxisomes are the main source of ROS in the light, while in darkness the mitochondria seem to be the main ROS producers. ROS are detected in virtually all intracellular organelles, as well as at the plasma membrane and, extra-cellularly, in the apoplast.

Environmental stresses including intense light, UV, temperature stress, heat/cold treatments, drought, and herbicides have all been demonstrated to increase ROS production [2,3]. In acute or prolonged treatments, the ROS generated can overwhelm the constitutive systems necessitating additional defenses. Thus, ironically, oxygen that it is essential for aerobic life—in its reduced forms—is one of the most toxic substances with which all organisms must cope. During the course of evolution, plants gained a high degree of control over ROS toxicity and are utilizing ROS as signal molecules for initiating defense mechanisms to sustain ROS homeostasis. Moreover, ROS play crucial roles in developmental processes in all living organisms [4]. These molecules are also important in plant responses to pathogens and immune defenses in mammals [5]. Controlling ROS toxicity, while modulating ROS as signals, requires a large gene network that in Arabidopsis is composed by at least 152 genes [6].

**Figure 1:**
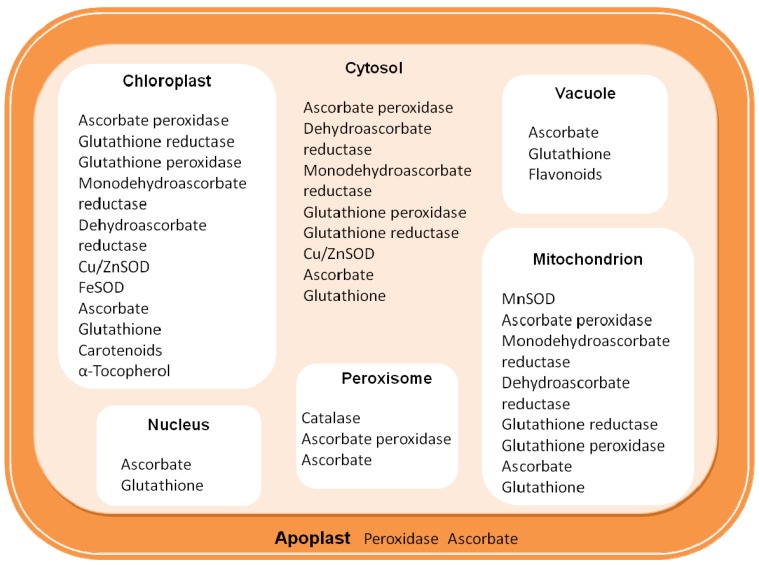
Distribution of the main antioxidant resources in plant cells.

Regulation of multiple redox reactions and ROS signals in plants requires a high degree of coordination and balance between signaling and metabolic pathways during a stress response in which cellular redox homeostasis of the cell is disrupted. This disturbance in equilibrium leads to sudden increase of intracellular level of ROS, and it has been estimated that 1%–2% of O_2_ consumed by plants is diverted to produce ROS in various subcellular loci. Given the challenge imposed by plant oxygen-evolving capability, each organelle or compartment has evolved mechanisms for the elimination of excess ROS accumulation (Figure 1).

ROS are not the only reactive molecules generated as by-products of enzymatic reactions. Recently, great attention has been paid to reactive nitrogen species (RNS) produced by a group of enzymes called nitric oxide (NO) synthases. As with ROS, the RNS cause biological damages because of their reactivity. Here, I will focus on ROS only.

## 2. Defense against ROS

ROS homeostasis in cells is reached at the balance between ROS production and ROS scavenging by antioxidant compounds and enzymes. Under normal growth conditions, the production of ROS in cells is low (240 µM s^−1^ O_2_^−^ and a steady state level of 0.5 H_2_O_2_ µM in chloroplast) [7] and ROS are normally in balance with antioxidant molecules. Non-enzymatic defenses include compounds of intrinsic antioxidant properties, water soluble such as ascorbate (vitamin C), glutathione, phenolic compounds and flavonoids, and lipid-soluble metabolites such as carotenoids and a-tocopherols (vitamin E). Antioxidants act as electron donors, reducing ROS to less harmful molecules; the oxidized products formed in the process are not very reactive or harmful. Purely enzymatic defenses include a variety of scavengers. Here, I have focused mainly on superoxide dismutase (SOD), catalase (CAT) ascorbate peroxidase (APX) and glutathione peroxidase, (GPX), which protect by directly scavenging superoxide radicals and hydrogen peroxide, converting them to less reactive species (Table 1). Homeostatic defenses can be overwhelmed in stress conditions, which increase ROS production (240–720 µM s^−1^ O_2_^−^ and a steady state level of 5–15 H_2_O_2_ µM) [7]. To reduce the damaging effects of ROS, aerobic organisms up-regulate both the non enzymatic and enzymatic antioxidant defenses [8].

### 2.1. Enzymatic Component of ROS Scavenging Defenses

#### 2.1.1. Superoxide Dismutase (SODs)

Superoxide dismutase (SOD: EC 1.15.1.1) belongs to a class of metalloproteins, which catalyze the dismutation of superoxide (O_2_^−^) into molecular oxygen (O_2_) and H_2_O_2_ [2O_2_^−^ + 2H^+^→O^2^ + H_2_O_2_]. The peroxide product must then be destroyed by CAT and/or peroxidases:
**SOD**O_2_^•−^ + O_2_^•−^ + 2H^+^→O_2_ + H_2_O_2_ (*K_2_* = 2.4 × 10^9^ M^−1^ s^−1^)**CAT**H_2_O_2_ + H_2_O_2_→2H_2_O + O_2_ (*K_1_* = 1.7 × 10^7^ M^−1^ s^−1^)**PX**H_2_O_2_ + R(OH)_2_→2H_2_O + R(O)_2_ (*K_4_* = 0.2–1 × 10^3^ M^−1^ s^−1^)


Thus, SOD and CAT serve in tandem as front-line antioxidant defenses. Therefore, it is not surprising to find that SOD and CAT are present in multiple subcellular locations (Figure 1).

Three classes of SODs can be distinguished on the basis of the metal co-factor at the catalytic site: Cu/ZnSOD, MnSOD, and FeSOD [10]. Plant cells generally contain Cu/ZnSODs in the cytosol and chloroplasts and possibly the extracellular space, FeSODs in chloroplasts, and MnSODs in the mitochondrial matrix and peroxisomes (Figure 1). Both Cu/ZnSODs and FeSODs are dimers, whereas MnSODs of mitochondria are tetramers. The analysis of deduced amino acid sequences suggests that the three types of SOD fall into two phylogenetic families: the Fe-MnSODs and the Cu/ZnSODs. Mn and FeSODs are the more ancient type; they probably evolved from the same ancestral enzyme, whereas Cu/Zn-SODs have no detectable sequence similarity to Mn and FeSODs. They might have evolved separately in eukaryotes [9] and have been selected in response to a common environmental stress: the oxygenation of the biosphere by photosynthetic organisms [10].

Unlike most other organisms, which have only one of each type of SOD in the different cell compartments, plants have multiple forms of each type encoded by more than one gene. The expansion of the gene families reflects the complex antioxidant defenses of plants. The three SOD isoenzymes are nuclear-encoded by small multigene families. SOD multigene families have been widely studied and several SOD genes have been cloned in a variety of plant species such as *A.* *thaliana* [11], maize [12], tobacco [13,14], tomato [15], and rice [16,17,18]. In the *A*. *thaliana* genome, the Cu/ZnSOD family includes three genes (*CSD1*, *CSD2* and *CSD3*), the FeSOD family includes three genes (*FSD1*, *FSD2* and *FSD3*), while only one gene encodes MnSOD [11].

In maize, the SOD multigene family consists of at least ten different isoforms: five are Cu/ZnSOD (*Sod1*, *Sod2*, *Sod4*/*Sod4A*, *Sod5* and *Sod9*), one is FeSOD (*SodB*) and four are MnSOD (*Sod-3*.*1*, *Sod-3*.*2*, *Sod-3*.*3* and *Sod-3*.*4*) [12]. The SOD gene family in maize seems more complex than it was earlier suggested and contains at least four additional not previously described genes: three FeSOD and one Cu/ZnSOD gene [19].

**Table 1:** Some natural antioxidants. Adapted from Scandalios [10].

Non enzymatic antioxidant molecules		Location	Primary ROS
Ascorbate (vitamin C)		Chl, Cyt, Mit, Per, Apo	H_2_O_2_, O_2_^•−^
Glutathione reduced (GSH)		Mit, Nuc, Per, Chl, Cyt, Apo Vac,	H_2_O_2_
β-Carotene		Chl,	^1^O_2_
α-tocopherol (vitamin E)		Cell and plastid membrane	ROOH, ^1^O_2_
Zeaxanthin		Chl,	^1^O_2_
**Antioxidant enzymes**			
Enzyme	EC number		
Superoxide dismutase (SOD)	1.15.1.1	Cyt, Apo, (Cu/ZnSOD);Chl, (Cu/ZnSOD; FeSOD); Mit, (MnSOD); Per, (Cu/ZnSOD)	O_2_^•−^
Ascorbate peroxidase (APX)	1.11.1.11	Chl, Cyt, Mit, Per, Apo	H_2_O_2_
Catalase (CAT)	1.11.1.6	Per	H_2_O_2_
Peroxidase (non-specific)	1.11.1.7	Cyt; CW	H_2_O_2_
Glutathione peroxidase (GPX)	1.11.1.19	Cyt, Mit,	H_2_O_2,_ ROOH
Glutathione reductase (GR)	1. 6.4.2	Mit, Cyt, Chl, Per	ROOH

Abbreviations: Apo, apoplast; Chl, chloroplast; CW, cell wall; Cyt, cytosol; Mit, mitochondria; Nuc, nucleus; ^1^O_2_, singlet oxygen; Per, peroxisome; Vac, vacuole.

In poplar, 12 genes were identified in the *Populus trichocarpa* genome (Phytozome) by BLAST using as queries *Arabidopsis* and poplar sequence functionally annotated as SODs in the NCBI data base. Seven SODs were classified as Cu/ZnSODs in three strongly supported sub-groups (PtCSD1, PtCSD2 and PtCSD3) corresponding to their putative *A*. *thaliana* orthologs. The MnSOD group is the smallest of the three, with two members, PtMSD1 and PtMSD2, derived from genome-wide duplication. The FeSOD group of *Populus* contains three members as in *Arabidopsis* [20].

Recently, molecular studies have evidenced that SOD gene expression can be changed by alternative splicing [21,22] and microRNAs [23]. Specialization of function among the different SOD isoenzymes could result from a combination of different subcellular locations and the features of gene regulation including promoter type and splicing.

To date, SODs have been characterized extensively for their physiological roles in protecting cells from toxic oxygen species in response to environmental stress such as ozone, drought, salinity, cold, *etc*. [13,24,25]. Increased transcript abundance is observed in response to abiotic and biotic stress to contrast oxidative stress exerts a significant role in stress tolerance. Transgenic plants over-expressing various SOD isoforms exhibit enhanced tolerance to oxidative stress and to various environmental stress factors; these results have been observed in many crops and model species including rice, potato, alfalfa, poplar, *Arabidopsis*, tobacco [25], *etc*. Moreover, SODs have been proved to be very sensitive to metabolic changes during cell life and respond to developmental stimuli. Induction of specific isoforms occurs in peroxisome during senescence [26,27], and transcription of MnSOD genes varies with both the ontogenetic stage and tissue growth rate [28,29].

Plant recalcitrance during *in vitro* culture might be associated with oxidative stress and ROS production. Strong induction of MnSOD and FeSOD enzymes was observed during somatic embryogenesis in horse chestnut [30], similarly high levels of SOD activity were necessary for embryogenic competence and development of somatic embryos of banana [31] and over-expression of Cu/ZnSOD enhanced *in vitro* shoot multiplication in transgenic plum [32].

#### 2.1.2. Catalase (CATs)

Catalase (CAT: EC 1.11.1.6; 2H_2_O_2_ = 2H_2_O + O_2_) enzyme is ubiquitous in the peroxisome, where it dismutates H_2_O_2_ into water and oxygen. Together with SOD and hydroxyperoxidases, CATs are part of a defense system for scavenging superoxide radicals [33]. The active CAT enzyme is a tetrameric iron porphyrin protein. In most eukaryotes, including mammals and several fungi, a single gene encodes the monomeric CAT subunits. In contrast, plant CATs are encoded by a small gene family, usually three or at most four isoenzyme genes in one species [34]. In *Nicotiana plumbaginifolia* [35] and maize [36], each CAT isoenzyme shows a distinct spatial and temporal pattern of expression throughout the entire life cycle of the plant. Different classes of CATs may be determined on the basis of their expression profile and a specific nomenclature has been adopted for this classification [37]. Class I CATs are expressed in photosynthetic tissues, synthesis is light-dependent, and the enzyme is involved in removal of H_2_O_2_. The class I includes the SU2 of cotton, *Cat1* of *N*. *plumbaginifolia*, CAT2 of *A*. *thaliana* [38], *Cat2* of *Zea mays* [39] and *Cat1* of *Prunus persica* [40]. Class II catalases are mainly found in vascular tissues and include the *Cat*2 of *N*. *plumbaginifolia*, *Cat*2 of castor bean [41], *Cat*A of rice a homolog to the maize *Cat*3 [42], a tomato catalase [43], *Cat*2*St* from potato [44], *CAT1* from *A*. *thaliana* [38] and *Cat2* of peach [40]. Class III CATs are mainly involved in the removal of H_2_O_2_ from glyoxysomes, and they are highly abundant in seeds and in young seedlings, while being almost absent at later stages of development (Table 2).

**Table 2:** Classification of plant catalases based on expression properties ^a^. Modified from Willekens *et al*. [37].

Species	Class I	Class II	Class III
*Gossypium hirsutum* (cotton)	SU2		SU1
*Nicotiana plumbaginifolia*	*Cat1*	*Cat2*	*Cat3*
*Ricinus communis* L. (castor bean)		CAT2	CAT1
*Zea mays* (maize)	CAT-2	CAT-3	CAT-1
*Arabidopsis thaliana*	CAT2	CAT1	CAT3
*Lycopersicon esculentum* (tomato)		TOMCAT1	
*Solanum tuberosum* (potato)		*Cat2St*	
*Prunus persica* (peach)	*Cat1*	*Cat2*	

^a^ Class I, expression in photosynthetic tissues, positively regulated by light; Class II, highest expression in vascular tissues; Class III, expression in seeds and young seedlings. Catalase names are reported as originally defined by the authors. Roman letters indicate a protein, and italics indicate a gene name.

**Figure 2:**
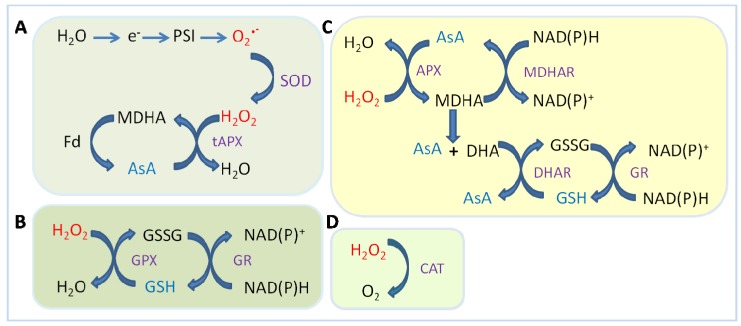
Different pathways for reactive oxygen species (ROS) scavenging in plants. Modified from Mittler [33]. (**A**) Water–water cycle (Mehler reaction); (**B**) Glutathione peroxidase (GPX) cycle; (**C**) Ascorbate–glutathione cycle; (**D**) Catalase (CAT). Superoxide dismutase (SOD) acts by converting O2^•−^ into H_2_O_2_, then ascorbate peroxidases (APX), glutathione peroxidases (GPX) and catalases (CAT) eliminate H_2_O_2_. In contrast to CAT, both APX and GPX require ascorbate (AsA) or glutathione (GSH) regenerating cycles that use electrons from the photosynthesis (A) or NAD(P)H (B, C) as reducing power. ROS are indicated in red, ROS-scavenging enzymes in violet and low-molecular antioxidants in blue. Abbreviations: CAT, Catalase; DHA, dehydroascorbate; DHAR, DHA reductase; Fd, ferredoxin; GR, glutathione reductase; GSSG, oxidized glutathione; MDHA, monodehydroascorbate; MDHAR, MDHA reductase; PSI, photosystem I; tAPX thylacoid bound APX.

Biochemical and molecular aspects of the complex roles of CATs have been thoroughly studied in herbaceous plants, both monocotyledons [36,39,42] and dicotyledons such as tomato [43], potato [44], cotton [45], *Nicotiana plumbaginifolia* [46,47], castor bean [48], barley [49], and *A*. *thaliana* [38]. In woody plants, the biochemical aspects of catalase have been studied in gymnosperms [50,51], horse chestnut [30], oak [52], and peach [40]. The spatiotemporal specificity that characterizes CAT isoform expression provided the rationale for using them as indicators of crucial developmental processes in woody species, such as somatic embryo conversion or rooting ability of microcuttings. In cypress, different catalase isoenzymes are linked to foliar dimorphisms of juvenile and mature shoots [51] and, similarly, in oak a specific catalase isoform was found specifically related to rooting, thus working as sensitive indicators of stress conditions during micro-propagation [52]. Plants use catalases in pathways other than those common to other eukaryotes. Catalase activity is a determining factor for the protection of photosynthetic cells against oxidative stress induced during abiotic stress conditions such as chilling, drought, salt and ozone [37,46,53]. Catalases (Figure 2D), together with ascorbate peroxidase, are a key enzyme in modulating the level of H_2_O_2_, which acts downstream of salicylic acid as a second messenger implicated in the signal transduction pathway that in plants leads to the development of systemic acquired resistance (SAR). The different affinities of the two enzymes for H_2_O_2_ (µm range for APX and mM range for CAT) suggest that they belong to two different classes of H_2_O_2_ scavenging enzymes: CATs might be responsible for removal of ROS during stress while APX might be responsible for the fine modulation of ROS during signaling [54].

#### 2.1.3. Ascorbate Peroxidase (APX)

Ascorbate peroxidases (EC: 1.11.1.11) catalyze the reduction of hydrogen peroxide to water by using ascorbate as the electron donor. Consequently, APX plays a role similar to that of CAT; however, the two enzymes exhibit distinctive features. APX utilizes ascorbate (AsA) as specific electron donor to reduce H_2_O_2_ to water with the parallel generation of monodehydroascorbate (MDHA), a univalent oxidant of ascorbate. MDHA is spontaneously disproportionated to AsA and dehydroascorbate (DHA). DHA reductase (DHAR) utilizes glutathione (GSH) as an electron donor to regenerate AsA from its oxidized form, DAsA (Figure 2C). Thus, the ascorbate–glutathione cycle located in the stroma of the chloroplast represents the main pathway to prevent the accumulation of toxic levels of H_2_O_2_ in photosynthetic organisms [55,56]. In addition to AsA–GSH, the water–water cycle, because of the electron flow from water in photosystem II (PSII) to water in PSI that occurs in this process across thylakoid membranes, participates in ROS detoxification and the dissipation of excess photon energy (Figure 2A).

Many studies reporting molecular cloning and physiological function of APX isoenzymes highlight the crucial role of APX in antioxidant protection in photosynthetic organisms. Furthermore, recent expression studies in response to stress conditions and pathogen attack indicate the importance of APX activity in intracellular signaling by controlling the H_2_O_2_ concentration. APX isoenzymes are distributed in four different cell compartments: soluble isoforms are found in the cytosol (cAPX), mitochondria (mitAPX) and chloroplast stroma (sAPX), while membrane-bound isoforms are found in peroxisomes and glyoxisomes (mAPX) and in chloroplast thylakoids (tAPX).

These enzymes are encoded by small gene families and expression of APX encoding genes is spatially and developmentally regulated and modulated by various environmental stimuli. Genomic and cDNA APX sequences are available from many plant species, showing that APXs are widely distributed in the vegetal kingdom. In the *A*. *thaliana* genome, the APX gene family includes nine genes encoding for isoenzymes found in the cytosol (APX1, APX2, APX6), chloroplast (APX3, APX4, APX6), mitochondrion (APX6, APX7), and peroxisome (APX3, APX5) plus one thylakoid-bound APX and one APX whose product is targeted to both chloroplast stroma and mitochondria [57]. In rice, the APX gene family includes eight members, namely: two cytosolic, two peroxisomal, two chloroplastic (stromal and thylakoid-bound), and two mitochondrial ones [58,59].

APX genes from spinach, pea, tomato, strawberry, and eucalyptus have been isolated and characterized. Particular attention has focused on the plant chloroplastic APX (tAPX) genes that are divided into two groups according the regulatory mechanism of expression. The first group comprises single genes from spinach (*Spinacia oleracea*), tobacco (*Nicotiana tabacum*), pumpkin (*Cucurbita* spp.), and ice plant (*Mesembryantheum crystallinum*) encoding two isoenzymes through a post-transcriptional alternative splicing regulation of the two final exons [60]. In the second group, single genes codify different isoenzymes, which are individually regulated. Genes from *A. thaliana*, rice and tomato belong to this group. APX plays a cooperative role in protection of each organelle. High and low temperatures, high light, drought and salt stress, heavy metals and pathogen attack modulate APX gene transcription [61]. The over-expression in transgenic plant of different APX genes has defined the specific action of single genes in stress tolerance. Transgenic rice plants over-expressing a cytosolic APX1 gene (*OsAPXa*), exhibited enhanced cold tolerance at the booting stage [62]. The over-expression in tobacco of a tomato chloroplastic tAPX gene was effective in minimizing photo-oxidative damage during high and low temperature stress [63,64]. The APX1 gene encoding cytoplasmic enzyme appears to be important in the acclimation of plants to combined stresses such as drought and heat [65]. Indeed, when cytosolic APX was over-expressed in tobacco chloroplasts, the plant was protected from several stresses, including osmotic [66]. The overexpression in transgenic tobacco of a peroxisomal ascorbate peroxidase poplar gene (*PpAPX*) improved plant drought resistance [67]. In transgenic tobacco plants, over-expression of the thylakoid-bound APX (*StAPX*) gene of tomato enhanced the tolerance to salt and osmotic stress [64]. *A*. *thaliana* mutants lacking cytosolic APX1 or tAPX and double mutants, *i*.*e*., *tylapx*/*apx1* were generated to study how different ROS signals, generated in different cellular compartments, are integrated in cells. The plants deficient in two APX genes were late flowering, and exhibited low protein oxidation during light stress and enhanced anthocyanin synthesis. These results suggest that the lack of conventional ROS removal enzymes such as cytosolic and chloroplastic APXs is compensated by a high degree of plasticity in ROS signaling and the existence of redundant pathways for ROS protection [68].

Other enzymatic defenses, such as peroxiredoxins (PRXs), which are a family of thioredoxin-dependent peroxidases, are also widely distributed in plant cells. They represent another antioxidant enzyme system that would join catalase and the ascorbate-glutathione cycle in the control of H_2_O_2_, particularly in the plant exposed to different types of biotic and abiotic stresses. In addition to their role in antioxidant defense mechanisms, PRXs also modulate redox signaling during development and acclimation. The most updated information on the structure and catalysis of Prx and their functional significance in plant metabolism has been recently reviewed by Dietz [69].

### 2.2. Antioxidant Molecules

Antioxidants inhibit the oxidation of other molecules and thus prevent them from causing oxidative damage. According to Halliwell and Gutteridge [70], mechanisms of antioxidant action can include: (1) suppressing ROS formation either by inhibition of enzymes or chelating trace elements involved in free radical production; (2) scavenging ROS and up-regulating antioxidant defenses [70]. Antioxidant molecules, which represent a second line of defense against ROS, include hydrophilic molecules (ascorbic acid, glutathione, phenolic compounds) and lipid-soluble metabolites (carotenoids and α-tocopherol) [24]. Ascorbic acid (AsA) and glutathione (GSH), which are coupled to each other through the ascorbate glutathione cycle, as above described, are major water-soluble antioxidants and Redox buffers in plant cells. These key metabolites, which are present at high (millimolar) concentration in chloroplasts and other cellular compartments, have also crucial functions in stress responses and organ development [71,72].

Ascorbic acid (vitamin C) is synthesized in the mitochondria and is transported to the other cell compartments through a proton-electrochemical gradient or facilitated diffusion. Ascorbic acid is considered as the most powerful plant cell antioxidant because of its ability to donate electrons in a number of enzymatic and non enzymatic reactions. It can also directly scavenge O_2_^•−^ and OH^•^ and regenerate oxidized carotenoids or α-tocopherol, thus providing membrane protection [24] and minimizing the damage caused by oxidative process through synergic action with other antioxidants. High concentration is maintained by an efficient recycling system that makes AsA a convenient antioxidant. Ascorbate is present in all plant tissues, generally being higher in photosynthetic cells, meristems, and in some fruits. The analysis of subcellular distribution of reduced and oxidized ascorbate in leaf cells of *A*. *thaliana* and *N*. *tabacum* was recently performed by high-resolution immune electron microscopy [73]. The nuclei and the cytosol of both plant species showed the highest concentration of ascorbate labeling, whereas mitochondria and plastids contained intermediate levels. The lowest ascorbate levels were found in the vacuoles. Lastly, the apoplastic ascorbate pool is important for control of cell elongation and triggering signal transduction cascades in response to external stimuli [74].

Glutathione is a thiol-containing tripeptide (glutamic acid-cysteine-glycine) that occurs in reduced form in all plant cell compartments: cytosol, endoplasmic reticulum, vacuole, chloroplast, mitochondria, and apoplast. It is the main non-protein thiol cofactor for various detoxifying enzymes and participates in several physiological processes such as the regulation of sulphur transport; the storage, detoxification of xenobiotics; signal transduction; and the expression of stress defense genes [72,75]. The main mechanisms controlling its concentration are the regulation of biosynthesis by γ-glutamylcysteine synthetase (γECS), the enzyme catalyzing the first step of glutathione biosynthesis [76,77,78]. GSH is essential for the antioxidant defense system particularly in chloroplasts, because it protects the photosynthetic apparatus counteracting the damaging effects of ROS. The cellular concentration of GSH varies considerably under biotic stresses and strong evidences correlate the ability of plant to withstand metal induced oxidative stress with high GSH concentrations [79,80].

GSH reacts chemically with a range of ROS while enzyme-catalyzed reactions link GSH to detoxification of H_2_O_2_ in the ascorbate-glutathione cycle (Figure 2C). Glutathione furthermore prevents denaturation of proteins caused by oxidation of thiol groups during stress. These functions results in the oxidation of GSH to form glutathione disulphide (GSSH). The ratio of GSH:GSSH is maintained by glutathione reductase (GR), which uses NAPDH to reduce GSSH to two GSH (Figure 2B,C). Beyond functioning in the ascorbate–glutathione cycle, GSH acts as direct electron donor to peroxides in reactions catalyzed by glutathione peroxidase (GPX). Unlike the constitutive GPXs of animals, plant GPXs are a large family of diverse isoenzymes, that are not constitutive but are stress induced; they do not contain selenium and catalyze GSH-dependent reduction of H_2_O_2_ at very low rates if compared to high rates of hydrogen peroxide production in plants [72]. Consequently, while APXs and CATs are predominant in H_2_O_2_ detoxification, GPXs may play a role in other parts of antioxidant metabolism, including the removal of lipid peroxides. GPXs generally increase in plants subjected to environmental constraints such as during fungal infection, water deflicit, and metal stress, and decreases during photo-oxidative stress. This finding demonstrates that GPXs are involved in the response to both biotic and abiotic stress conditions [81].

**Figure 3:**
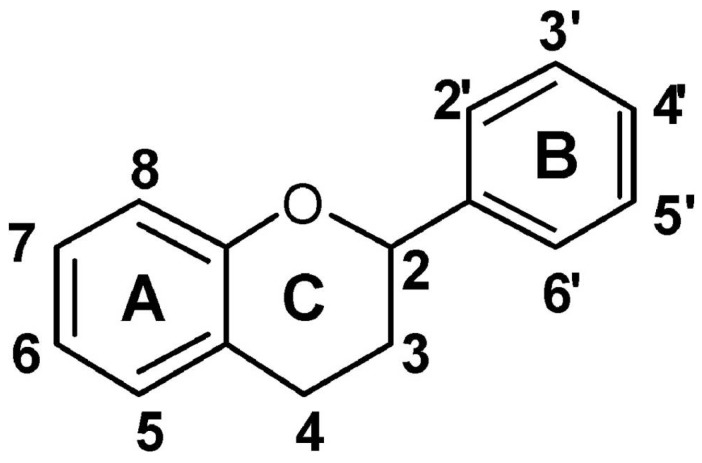
Basic structure of flavonoids.

Among the many antioxidant molecules, flavonoids and carotenoids are particularly interesting because they are common in fruits and relevant for human health. Phenolic compounds represent an important class of water soluble antioxidants. They are widespread, often at high levels, in almost all plant foods as flavonoids, tannins, and lignans. The most highly studied of these compounds are the flavonoids—a large class of more than 9000 structurally diverse compounds—which are synthesized by plants via the phenylpropanoid pathway. They include chalcones, flavones, flavonols, flavanones, anthocyanins, and isoflavonoids [82]. The flavonoid basic structure is relatively simple and consists of 15 carbon atoms arranged in two aromatic C_6_ rings connected by a C_3_ unit; the rings are labeled A, B and C (Figure 3).

Flavonoid biosynthesis has been intensively studied in genetically tractable species as maize, snapdragon, and petunia; all the genes encoding the biosynthetic enzymes and transcription factors have been isolated and characterized. Many studies have been performed to verify the relationship between flavonoid structure and activity as ROS scavenger. Radical scavenging activity relies on the structure and the substituent of the heterocyclic ring. The catechol structure in the B ring, in particular, is the major determinant for flavonoid scavenging capacity [83].

**Figure 4:**
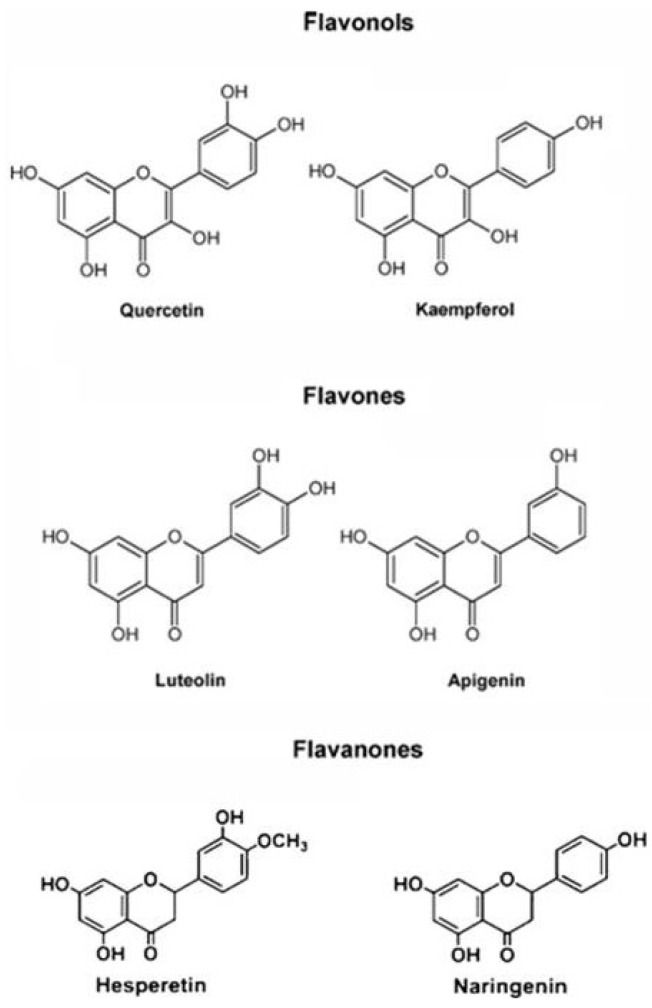
Structures of flavonoids showing differences in B-ring hydroxylation within subclasses.

Flavonoids commonly occur in plants, and many are glycosylated derivatives stored in the vacuole; besides vegetables and fruits, these compounds are found in seeds, nuts, grains, spices and different medicinal plants. The flavones apigenin and lutoelin are found in cereals and aromatic herbs, while the flavanones including hesperetin and naringenin are mainly found in citrus fruits. The flavonols quercitin and kaempferol are widespread in vegetables and in the skin of fruits (Figure 4). Antocyanidins and their glycosides antocyanins are abundant in berries and red grape.

Distribution among plants, structural aspects and antioxidant activity of most common flavonoids are extensively reviewed by Pietta [84]. Flavonoids are involved in the resistance to plant pathogens and also provide a number of unrelated functions in the ecology of plants. Because of their attractive colors, flavones, flavonols and anthocyanidins play an important role in the attraction of pollinator insects and fruit eating animals. The catechins and other flavanols are bitter and may play a role in defence mechanisms against insects and predators, and furthermore they act as scavengers of ROS produced by the photosynthetic electron transport system in plant cells.

Several flavonoids proficiently chelate trace metals that play a role in oxygen metabolism. Many flavonoids biosynthetic genes are induced under stress conditions. Drought, chilling, wounding, metal toxicity and nutrient deficiency increase flavonoids levels [85]. Moreover, due to their favorable UV-absorbing capacity, flavonoid compounds scavenge UV-generated ROS and protect plants against harmful UV radiation by absorption of this harmful radiation [86].

**Figure 5:**
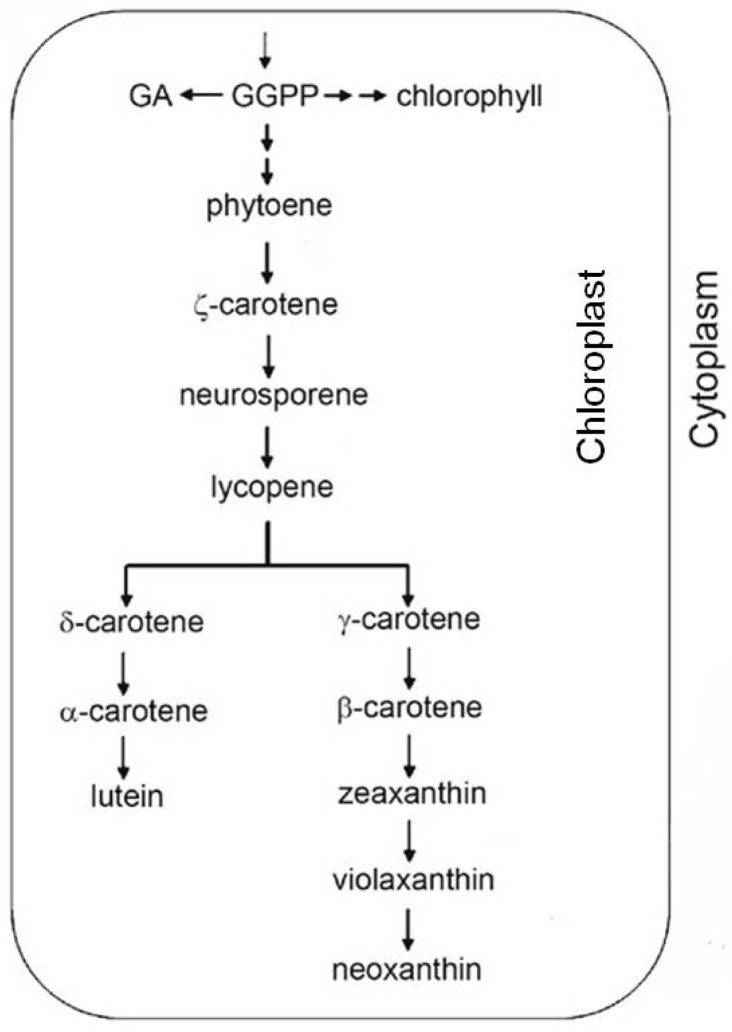
Carotenoids are the accessory pigments ubiquitous in photoautotrophs. These pigments participate in light-harvesting, fulfill photoprotective functions, and stabilize the pigment-protein complexes of the photosynthetic apparatus.

Carotenoids, which represent the main lipophilic antioxidants, are isoprenoid-derived molecules synthesized by plants, bacteria, fungi, and prokaryotic algae. Carotenoids are 40-carbon isoprenoids with polyene chains that may contain up to 15 conjugated double bonds. Based on their composition, carotenoids are divided into two classes: carotenes contain only carbon and hydrogen atoms while xanthophylls are the oxygenated forms of carotenes [87]. Plant carotenoids are generally synthesized and located in plastids, and their production is driven by nuclear-encoded enzymes. The synthesis in plants follows this biosynthetic sequence: phytoene→phytofluene→ζ-carotene→neurosporene→lycopene→α-carotene and β-carotene (Figure 5).

Each enzymatic step from phytoene to lycopene adds one double bound to the molecule. Lycopene is a symmetrical molecule containing 13 double bonds. Enzymatic cyclization of lycopene marks a pathway branching point: one branch leads to β-carotene and its derivative xanthophylls, while the other leads to α-carotene and lutein [87]. The carotenoid biosynthetic pathway has been extensively studied, and all the genes encoding the biosynthetic enzymes have been isolated and characterized from various organisms [88].

There are hundreds of carotenoids and most of them exhibit antioxidant activity. The best documented antioxidant actions of carotenoids are their abilities to quench singlet molecular oxygen and to trap peroxyl radicals. The antioxidant activity of carotenoids is influenced by the pattern of conjugated double bonds in the polyene backbone. In fact, it is the number of conjugated double bonds present in the molecule that determines the efficiency for physical quenching of ^1^O_2_. β-Carotene, zeaxanthin, cryptoxanthin, and α-carotene belong to the group of highly active quenchers of singlet molecular oxygen [89].

Because of its importance in human health by preventing a range of diseases, the bioavailability of provitamin A carotenoids and their bio-efficacy have been extensively studied. In particular, stable isotope methodology has advanced our knowledge. Presently, the relative mean conversion factors of β carotene into vitamin A by mammals are calculated by weight as 12:1 for orange fruit and that of β carotene from vegetables as 26:1. Nevertheless, the food matrix plays an important role in carotenoid bioavailability. For example, a certain amount of fat is needed to ensure the absorption of fat-soluble provitamin A. Furthermore, a large variation in bioconversion of dietary β-carotene was observed in relation to genetic variability in β carotene metabolism on individual human subjects [90].

Approximately 50 carotenoids are called “provitamin A” compounds, because the body can convert them into retinol, an active form of vitamin A. These (particularly β-carotene) represent the major dietary source of vitamin A. β-Carotene and lycopene have been best studied because they are the most common carotenoids in fruits and vegetables. These pigments cover a wide range of functions: photo-protective function against oxidative damage during photosynthesis, contribution to plant cross-talk with symbiotic organisms, and modulation of interactions with pests and pathogens [91].

More than 800 carotenoid species with a linear or cyclic structure have been discovered in plants. The carotenoid composition varies from species to species, and the accumulation of specific carotenoids in fruit and flower chromoplasts is a highly, developmentally regulated process [92]. Consequently, their concentrations in fruits and vegetables vary with plant variety, degree of ripeness, time of harvest, growing and storage conditions. The orange-colored fruits and vegetables including carrots, pepper, apricots, mangoes, squash, papaya, and sweet potatoes contain significant amounts of β-carotene, α-carotene, and β-cryptoxanthin. Green vegetables, especially spinach, contain β-carotene and are the best sources of lutein. Lycopene produces the red color of tomatoes, and significant carotenoid variations are observed during fruit ripening [93].

Tocopherols are lipid-soluble antioxidant synthesized by all plants. They protect lipids and other membrane components by scavenging and quenching various ROS and lipid by-products of oxidative stress. Out of four isomers of tocopherols present in plants, α-tocopherol has the highest antioxidant activity and represents the major vitamin E compound; it is the only tocopherol absorbed efficiently by humans. α-Tocopherol is located in the chloroplast envelope and thylakoid membranes; high levels have been found in leaves of many plant species. Moreover, given that α-tocopherol increases membrane rigidity, its concentration, together with that of the other membrane components, may be regulated to afford adequate fluidity for membrane function. α-Tocopherol levels change significantly during plant growth and development, and in response to environmental stress. A number of reports deal with the increase in tocopherol in relation to chilling tolerance and water stress. Recently, activation of synthesis during oxidative stress was demonstrated to reflect activation of the pathway related genes. Furthermore, recent studies on tocopherol-deficient plants indicate that α-tocopherol may affect cellular signaling in plants [94,95,96,97].

Ascorbic acid, flavonoids, carotenoids, and tocopherols, because of their antioxidant properties and health promoting effects, represent attractive targets for bio-fortification programs. The increase of specific antioxidant products, both with molecular or conventional approaches, is an important and interesting topic for plant biotechnology and breeding. A variety of genetic strategies can be used to modify plant metabolism to enhance the availability of these compounds. The most popular are the over-expression of a known rate-limiting enzyme or the repression of a competitive pathway to canalize flux toward the desired metabolite. Furthermore, bioaccumulation (the amount of a particular nutrient that can be accumulated in plant tissues) represents an important but not ultimate goal. In fact, bioavailability (the amount that can be absorbed after consumption) of nutrients in crops is a more important indicator of its nutritional quality. Reducing anti-nutritional compounds and/or increasing the quantities of nutritional enhancers can therefore increase the bioavailability of nutrients. The bio-fortification strategies, which exceed the aims of this article, have been recently reviewed by Zhu and co-workers [98].

## 3. Antioxidant System during Development, Maturation and Fruit Ripening with Particular Attention to *Prunus* and *Citrus* spp.

The critical role exerted by ROS in processes involved in protecting plants from biotic and abiotic stresses has been the subject of extensive studies. Moreover, ROS are also acknowledged as important regulators of plant growth and development. For example, proper maintenance of ROS homeostasis is a crucial step for successful regeneration in the early stages of shoot organogenesis in sweet orange (*Citrus sinensis*) [99]. There is a tremendous range of diverse processes in which ROS have been implicated acting as regulators of cytoplasmic signaling or as modulators of cell wall properties [100,101,102,103]. Recent insights into ROS biogenesis in a developmental context are discussed by Swanson and Gilroy using as examples the elongation of the root and the tip growing systems [29].

Fruit ripening, maturation, and senescence are developmental processes strongly related to agricultural production and are consequently of great interest to both crop physiologists and geneticists. Fruit development and ripening are dynamic events that involve a complex series of molecular and biochemical changes that result in modifying the complex network of metabolites and proteins. Biochemical processes include the degradation of chlorophyll and starch, the biosynthesis of pigments and volatile compounds, the accumulation of sugars and organic acids, as well as cell wall softening [104,105]. Nowadays, particularly through proteomic and metabolomic analysis during ripening stages, the first high-resolution picture of the metabolic dynamics during fruit development is available. Differential proteomic studies at different ripening stages, from immature to mature fruits, at the level of the whole fruit, but also with isolated flesh, seeds and skin, have been accomplished. Both climacteric fruit as tomato (*L*. *esculentum*) [106,107], peach (*P*. *persica* L. Batsch) [108,109,110,111,112,113,114], apple (*Malus domestica*) [115] and non-climacteric fruits, as grape (*Vitis vinifera*) [116,117,118,119,120,121,122,123,124], strawberry (*Fragaria* spp.) [125,126] and *Citrus* spp. [127] have been investigated with proteomics. The combination of protein separation methods (2DE, 1-DE, LC, DIGE and 2-DE plus western blotting) accompanied by MS identification provides a dynamic picture of fruit biology [128]. These investigations offered significant information on molecular components involved in fruit ripening, including sugar metabolism, ethylene biosynthesis and perception, cell wall depolymerisation, signal transduction, stress responses, and enhanced accumulation of reactive oxygen species (ROS). Fruit ripening is an aerobic process generating ROS; these molecules initiate and enhance degenerative processes associated with fruit maturation, which is considered a protracted form of senescence [129]. During ripening and senescence, some of the changes in the physical and chemical characteristics of fruit are not so easily observable, but impact fruit quality, while others, in addition to the perceivable alterations in fruit aroma, pigmentation, texture, and sweetness [108]. Proteomic studies are contributing a deeper understanding of fruit maturation also in relation to post-harvest quality and shelf-life feature of the fruit. This is particularly important for fleshy fruits, such as peaches and apricots that are extremely susceptible to handling and physical injuries and require a long storage period before consumption of the ripe fruit. During post-harvest, the proteins related to stress response, cellular homeostasis, carbohydrate and amino acid metabolism were affected and some of them changed after low temperature storage [109]. Ripening and senescence result in ROS accumulation and changes in antioxidant activity in climacteric fruits, *i.e.*, tomato [130] and peach [131], characterized by a burst of ethylene production and respiratory activity. The involvement of ROS in ripening and post-harvest senescence in peaches was confirmed by studies using 2-DE and immunoblotting performed with isolated mitochondria. The results indicated that oxidative damage to mitochondria caused by ROS resulted in protein carbonylation of specific mitochondrial proteins such as MnSOD. The oxidative modification and loss of catalytic activity of the antioxidative enzyme, in turn, facilitated the release of superoxide radicals in mitochondria thus linking oxidative damage to fruit senescence [129]. In orange (*C*. *sinensis*), several protein changes characterize stage III (the ripening and maturation stage). In this later stage of development, beside the many proteins related to sugar metabolism, those involved with ROS metabolism including GSH, PX, GST, CAT, SOD and APX were also increased [128]. This last enzyme participates in the metabolism of ascorbate (vitamin C), an important constituent of orange fruits [132]. Proteins related to antioxidant defense were also found particularly expressed in ripening blood sweet oranges (*C*. *sinensis* cv. Moro), which are characterized by red pigmentation of flesh and rind, in comparison with the common cultivar, highlighting their value for human health and nutrition [133].

The study of antioxidants in fruits is important for several reasons. Fruits are a significant part of our daily diet and increasing fruit consumption is an easy and logical strategy to increase antioxidant intake and decrease oxidative stress. Adding antioxidants to the human diet could reduce diseases such as cancer. The growing interest in nutraceuticals and functional foods has driven plant breeders to consider selection of crops with higher antioxidant contents in various common fruits. In addition to their relevance in human health, antioxidants also contribute in the protection of fruit tissues from potentially toxic ROS [24], thus increasing crop stress tolerance.

Fruits contain many different antioxidant components in different mixture according the species. Vitamin C, vitamin E, or β carotene and phenolic compounds, in particular flavonoids, constitute the primary antioxidant resources of fruits. Therefore, it was of enormous interest to measure the content of bioactive compounds and the total antioxidant capacity in a fruit or vegetable. Consequently, studies on commonly consumed fruits supply information useful for breeders, nutritionists, and consumers [134,135,136,137]. Several methods have been developed to assess “total” antioxidant activity of fruits; these assays differ in their chemistries and sensitivity. A common assay is iron-based assays FRAP (ferric reducing antioxidant power) [137]. ABTS^+^ [azino-bis (3-ethyl-benzothiazoline-6-sulfonic acid)] assay and DDPH (1,1-diphenyl-2-picrylhydrazyl), which measure free radical capacity, are more sensitive than the iron-based assay [138,139,140]. The results, even if obtained with different methodologies, indicate that antioxidant capacity and the concentrations of bioactive molecules differ greatly among the fruit species. Variability in antioxidant composition exists also among and within cultivars, and it is influenced by climatic conditions, agronomic practices, and pre- and post-harvest conditions [141]. Antioxidant capacity is generally associated with total phenolic content, which is likely a reasonable predictor for the cellular antioxidant activity of fruits [136]. Nevertheless, it has been also suggested that if phenolic compounds could be one of the main components responsible for reducing capability, they could not be main components in free radical scavenging in the fruits analyzed. Because of synergic actions of the different molecules on antioxidant activity, the total content of bioactive compounds is now taken into account by crop breeders in addition to overall fruit quality and organoleptic properties, traditionally used for the selection of new cultivars. Peaches and oranges merit particular attention in this context, because of the importance that these species have for human nutrition and health in addition to their economic relevance for Mediterranean countries and in the Americas. Oranges and peaches plus nectarines are, after apples, the most important fruit crops in Europe with a production in 2011 of 6,273,864 and 4,329,917 tons, respectively [142]. Peaches and nectarines have a lower antioxidant capacity than other fruits including strawberry or orange, however, they are nutritionally important because they are a popular summer fruit and two of the mostly widely consumed fruits. Fresh fruits are an excellent source of sucrose, citric and malic acids, carotenoids and lactones, as well as polyphenol and pectic substances, which determine fruit sensory quality and nutritive value [143,144]. The analysis of antioxidant systems and total content of antioxidant molecules—flavonoids, anthocyanins, carotenoids, and vitamin C—has been performed on ripe fruit of peach and nectarine cultivars commonly grown all over the world [145,146,147,148,149,150]. The results obtained with comparable methods are presented in Table 3.

**Table 3:** Antioxidant molecule contents and relative antioxidant capacity (RAC) in fruit flesh of peach.

Compounds	Means ± SE	
	Abibi *et al.* [145]	Cantin *et al.* [142]
Vitamin C	4.0 ± 0.1	3.7 ± 1.0
Total phenolics	32.6 ± 0.7	36.4 ± 11.0
Flavonoids	12.5 ± 0.6	8.8 ± 0.4
Anthocyanins	3.2 ± 0.2	3.0 ± 0.3
RAC	464.2 ± 12.5	405.0 ± 4.9

**Figure 6:**
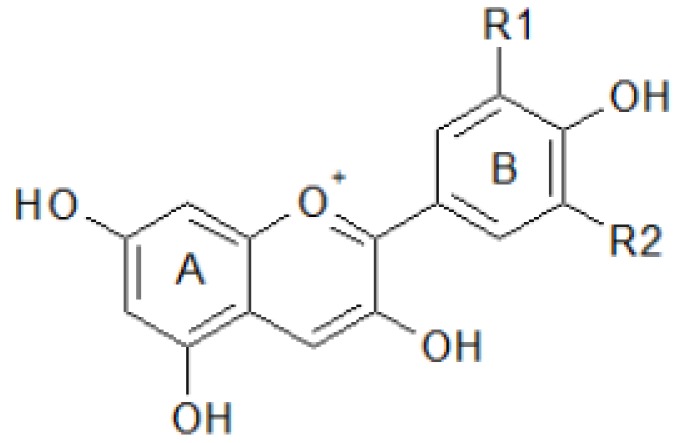
Anthocyanin structure.

Anthocyanins (Figure 6) generate the red color present on skin and in flesh strongly characterizing peach and nectarine fruit. In addition to their contribution to aesthetics, they have strong antioxidant properties in relation to their different hydroxylation and glycosilation [83]. The main anthocyanins present in peach are cyanindin-3-glucoside and cyanidin-3–rutinoside [151].

Phenolic distribution is an important aspect of the overall phenolic composition and antioxidant capacity. These compounds are not uniformly distributed within the fruit tissues, being mainly located in the epidermis and sub-epidermis layers of the fruit [152]. For example, in a detailed study of Californian stone fruits, Gil and co-workers [145] gave a complete picture of antioxidant capacity and content in phenolics, carotenoids and vitamin C, both in flesh and peel of the ripe fruit (Table 4). This study and those performed by scientists of other countries on different local varieties demonstrated the great variability existing in term of antioxidant capacity among the cultivars.

Phenolics are the only compounds that correlate with total antioxidant capacity; both hydroxycinnamic acid and flavan-3ols are strongly correlated to total phenolic levels. In contrast, anthocyanins and flavonols are primarily in the peel and are therefore not correlated to total phenolic contents. Considering all available data, the antioxidant capacity of a fruit is the result of synergism among the diverse mixture of antioxidant molecules reflecting cultivar and genotype. Camejo and co-workers studied the oxidative parameters and antioxidant enzymatic system, in particular the activities of SOD and the components of the ascorbate-glutathione cycle. They utilized peach fruits suitable for fresh consumption and for the processing industry at two maturation stages [147]. An increased activity of APX, MDHAR, and GR and changes in SOD isoenzyme patterns were observed in mature fruits, illustrating the efficacy of the antioxidant system to cope with the oxidative process accompanying ripening.

The increase in protein, antioxidants, and activities of enzymes involved in antioxidant metabolism together with the decrease of oxidants including both superoxide radical (O_2_^•−^) and hydrogen peroxide (H_2_O_2_) support the idea that synthesis of antioxidants or redox modulators is induced during ripening to protect the fruit against oxidative damage that might otherwise result during fruit development and maturation. On the other hand, the antioxidant system greatly contributes to delaying the senescence process in harvested fruit. Similar analysis during orange pulp fruit ripening and maturation performed demonstrated a different dynamics in the antioxidant system [153]. In fact, fruit maturation and ripening were accompanied by a general decrease of antioxidant enzyme activities (SOD, CAT, APX and DHAR, except GR) and by a decrease in both the reduced and oxidized forms of ascorbate and glutathione. The decline in the antioxidant system paralleled the decline of antioxidant activity (FRAP values) and consequently was correlated with increased oxidative stress associated with ripening of sweet orange fruits.

**Table 4:** Antioxidant molecule contents in ripe fruit (flesh and peel) of peach and nectarine differing for flesh color. Modified from Gil *et al.* [145].

Compounds	Content range	
Ascorbic acid vitamin C mg/100g fresh weight	Nectarines white flesh	5–14
	Nectarines yellow flesh	5–7
	Peaches white flesh	6–8
	Peaches yellow flesh	4–13
Total phenolics mg/100g fresh weight	Nectarines white flesh	14–102
	Nectarines yellow flesh	18–54
	Peaches white flesh	28–111
	Peaches yellow flesh	21–61
Carotenoids	Nectarines white flesh	7–11
µg/100g fresh weight	Nectarines yellow flesh	80–157
	Peaches white flesh	8–17
	Peaches yellow flesh	95–197

**Figure 7:**
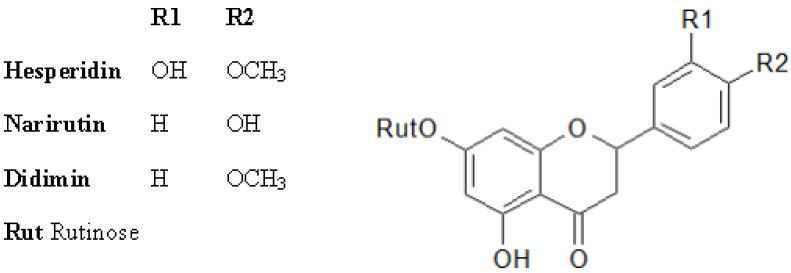
Chemical structures of the flavanones in oranges.

In *Citrus* fruit, beside the ROS scavenging enzymes, the non-enzymatic components included carotenoids, polyphenols, and vitamin C [154,155,156]. Carotenoids are the pigments responsible for the external and internal coloration of fruit of most citrus species; therefore, their content and composition have a strong impact on both the commercial and nutritional quality of the fruit. Differences in carotenoid contents and profiles were observed among tissues and species. The main carotenoids present in citrus are β-cryptoxanthin, lutein, zeaxanthin, lycopene, β-carotene, and α carotene. The presence of lycopene characterizes some citrus mutants such as the Star Ruby grapefruit and the orange varieties Vaniglia Sanguigno, Cara Cara, and “Hong Anliu”. Lycopene confers bright red coloration and superior carotenoids contents to the pulp compared to non mutant fruits [157]. The most important flavonoid classes are the flavanones (Figure 4) and the anthocyanins (Figure 6), the latter present specifically and exclusively in red blood oranges. The most significant flavanones are hesperidin, narirutin, and didimin (Figure 7) whose contents in blood oranges are reported to be almost two to three times higher than blond oranges [158,159]. Anthocyanins are evident in rind, leaves and flowers of some citrus (as lemon and *Papeda citrus*) only during the first developmental stage. In citrus mature fruits, anthocyanins are exclusively expressed in red-blood orange, which are the Tarocco, the Moro (both native to Italy), and the Sanguinello (native to Spain) and their hybrids in which anthocyanins production is strictly linked to genotype and environmental conditions [160]. In the blood orange juices, the most widespread anthocyanins are cyanidin-3-glucoside and cyaniding 3-(6″-malonyl) glucoside [161,162,163].

Vitamin C is one of the main nutrients in citrus fruit and their products are one of the principal suppliers of dietary vitamin C. Many factor influence vitamin C content in the fruit: the species and cultivar, rootstock, climate condition, maturity state and position of fruit on the tree, production factors, and harvest and storage conditions. Thus, because of many horticultural and climatic variables involved in growth, it is not surprising that wide ranges of vitamin C levels were reported in the different citrus fruit. On the basis of the data available, oranges are richest in vitamin C, followed by grapefruit, lemons, tangerines and limes. The approximate ranges for vitamin C of mandarin, expressed as mg/100 mL of juice, are 15–55 (tangerine, USA), 20–60 (Mediterranean) and 20–50 (Satsuma, Japan). In lemons and limes, vitamin C values in juice show ranges of about 20–60 mg/100 mL and about 15–45 mg/100 mL, respectively. The vitamin C content of grapefruit has a range from 25 to 60 mg/100 mL of juice. The wide vitamin C ranges might reflect that lemons and limes are genetically complex biotypes and are regarded as introgressed trihybrids. In oranges, differences in vitamin C level, ranging from 30 to 88 mg/100 mL of juice has been observed among varieties, but also seasonal variations were described. Orange juices from January to March are richer in vitamin C than juices from April to July [164]. Furthermore, bioavailability of vitamin C is enhanced by co-presence with bioflavonoids. In fact, although natural and synthetic Vitamin C is chemically identical, the ascorbate in the citrus extract was found to be more bioavailable in human subjects [165]. Because of the high amounts of vitamin C, anthocyanins and carotenoids in blood orange, particular attention was paid to antioxidant properties of the juice. *In vivo* studies conducted on healthy people have shown that red orange juice consumption determines a significant increase in plasma vitamin C beta-cryptoxanthin, zeaxanthin, and cyanidin-3-glucoside. These results suggest that blood orange juice, due to synergic effects of its compounds, represents a bioavailable source of antioxidants that positively affect the antioxidant defense system [166]. As a consequence, special attention has been paid by Italian researchers to evaluation of the stability of ascorbic acid in red pigmented blood orange juice and its detrimental effect on color stability. In blood orange juices, vitamin C ranges between 40 and 80 mg/100 mL and in Tarocco cultivar it is about 70–80 mg/100 mL [167]. Fruit storage for long periods could decrease vitamin C content. Lower ascorbic acid content was observed after 65 days in Tarocco and Moro oranges without compromising antioxidant properties of the fruits; hence, these cultivars represent an excellent source of phytochemicals with potential health benefits for dietary supplement. 

## 4. Conclusions

Antioxidant defense machinery involved directly or indirectly with ROS scavenging in plants represents a powerful tool to counteract oxidative stress at the cellular level. These antioxidant systems, which are essential for plant defense to avoid damage from abiotic and biotic stress, also represent a valuable resource for human health. In fact, although we cannot live healthily forever, we can help our cells to ameliorate oxidative stress and its detrimental effects through consumption of natural antioxidant derived from plants. More than ever, consumers desire attractive and tasteful foods that are safe, natural, and health-promoting. The natural antioxidants present in fruits are the easy, pleasant and logical strategy to increase antioxidant intake simply through increasing fruit consumption in the daily diet. 
