# Correction: Racchi, M.L. Antioxidant Defenses in Plants with Attention to *Prunus* and *Citrus* spp. *Antioxidants* 2013, *2*, 340-369

**DOI:** 10.3390/antiox3010189

**Published:** 2014-03-20

**Authors:** Milvia Luisa Racchi

**Affiliations:** Department of Agri-Food Production and Environmental Sciences, Section of Agricultural Genetics–DISPAA, University of Florence, via Maragliano 77, Firenze 50144, Italy; E-Mail: milvia.racchi@unifi.it; Tel.: +39-055-275-5537

I have found two inadvertent errors in my review published in Antioxidants [1].

- On page 355, text line 33 and again on page 356 text line 3 in the legend of Figure 6, the word Anthocyanins should be amended to Anthocyanidins.

I apologize to the readers.
